# Physical activity and sedentary behavior among school-going adolescents in low- and middle-income countries: insights from the global school-based health survey

**DOI:** 10.7717/peerj.17097

**Published:** 2024-04-24

**Authors:** Hui Li, Wenyu Zhang, Jin Yan

**Affiliations:** 1 College of Physical Education and Health Sciences, Zhejiang Normal University, Jinhua, China; 2 Foundation Department of Education, Shandong Communication and Media College, Jinan, China; 3 School of Physical Education and Sports Science, Soochow University, Suzhou, China; 4 Centre for Active Living and Learning, College of Human and Social Futures, University of Newcastle, Newcastle, NSW, Australia

**Keywords:** Physical activity, Sedentary behavior, Adolescents, Survey, Low and middle-income countries

## Abstract

**Background:**

The Global School Student Health Survey (GSHS) is being carried out by students in various countries across the globe to advance improved health programs for youth. However, in comparison to high-income countries, adolescents in low- and middle-income countries (LMICs) are generally at an early stage of understanding regarding physical activity (PA) and sedentary behavior (SB), often exhibiting low levels of PA and high levels of SB. Furthermore, there is limited evidence connecting PA and SB in school-going adolescents from LMICs.

**Purpose:**

The objective of this review was to synthesize the available evidence regarding PA and sedentary behavior among school-going adolescents in LMICs using data from the GSHS.

**Method:**

On March 18, 2023, a systematic literature search was performed across four electronic databases, namely Web of Science, PubMed, ScienceDirect, and EBSCO with n odaterestrictions. Studies were eligible if they: (1) utilization of data sourced from the Global Student-based Health Survey; (2) exploration of physical activity; (3) specific focus on adolescents; (4) conducted in low- and middle-income countries; (5) study design encompassing observational; (6) published as English journal articles.

**Results:**

Among the 29 studies included in the analysis, the majority revealed elevated levels of sedentary behavior and diminished levels of PA in low- and middle-income countries. Furthermore, notable disparities in physical engagement and sedentary behavior were noted between male and female adolescents (*p* < 0.001). Augmented PA among teenagers was observed to correlate with higher consumption of vegetables and fruits (AOR = 1.30; 95% CI [1.13–1.50]; *p* < 0.001), decreased alcohol consumption, and a reduced prevalence of loneliness and depression (aOR 1.37, 95% CI [1.18–1.59]).

**Conclusions:**

The results of this review affirm that in contrast to high-income countries, adolescents in low- and middle-income countries (LMICs) are in the early stages of comprehending physical activity, marked by low levels of PA. Physical activity and sedentary behavior in school-going adolescents from LMICs appear to be influenced by factors such as policies, cultural norms, socioeconomic conditions, as well as gender, and age.

## Introduction

Regular participation in physical activity (PA) generates many health benefits, such as improved muscular and cardiorespiratory fitness (Zheng et al., 2023), healthy bone development (Cox et al., 2020; Fan et al., 2022; Shi et al., 2022), improved well-being (*e.g*., self-perception, self-esteem, and happiness), and a reduced incidence of ill-being (*e.g*., depression, anxiety, stress) (Liu et al., 2023; Mavilidi et al., 2021). The World Health Organization (WHO) suggests that children and adolescents should accumulate at least 60 min of moderate- to vigorous-intensity PA daily (Bull et al., 2020; Chaput et al., 2020). In contrast, sedentary behavior (SB) has been linked to negative health outcomes such as obesity, cardiovascular disease, and poor mental health (Chen & Yan, 2020; Liu et al., 2022; Shen et al., 2020; Tremblay et al., 2016). The duration of SB is positively correlated with digestive function and cardiovascular disease risk in adolescents (Jorde & Grimnes, 2011). Cabanas-Sánchez et al. (2019) noted that adolescents with more PA interventions had a greater degree of prosocial behavior and that SB was not conducive to adolescents’ emotional regulation or teamwork. Similarly, Chaput et al. (2020) discussed the 24-h movement guidelines for Canadian youth and concluded that integrating PA with SB and sleep avoids the limitations of focusing only on exercise behavior and that this fundamental shift lays the foundation for the overall well-being of adolescents. School-aged children and youth with high PA, high sleep, and low SB generally have more desirable markers for obesity and cardiometabolic health than children and youth with a combination of low PA, low sleep, and high SB (Saunders et al., 2016; Tremblay et al., 2016).

When national income levels are taken as a starting point, sitting time is more strongly associated with compounding outcomes for people in low- and middle-income countries (LMICs) than for those in high-income countries (Darfour-Oduro et al., 2018). Compared with individuals who reported sitting for less than 4 h/day and having high levels of PA, participants who sat for eight or more hours per day had a 17% to 50% greater risk of comorbid outcomes across all levels of PA, and this risk decreased as PA levels increased (Lear et al., 2017). Previous studies have shown significant variations in PA levels across different regions, with relatively low rates of physical inactivity observed in some countries, such as Myanmar (74.8%), and rates as high as 90.7% in Cambodia (Peltzer & Pengpid, 2016). These differences may stem from various factors, such as culture, infrastructure, education level, and socioeconomic conditions (Darfour-Oduro, Andrade & Grigsby-Toussaint, 2020). This understanding aligns with the perspective of Chen et al. (2016), who suggested that urbanization may prompt lifestyle changes that lead to decreased levels of PA. Furthermore, several studies have demonstrated that age, gender, and family economic status may influence participation in physical activity (Manyanga et al., 2014). Koyanagi, Stubbs & Vancampfort (2018) speculate that this may be because sitting is generally associated with higher socioeconomic status and better-paying jobs in high-income countries. Similarly, Barbosa Filho et al. (2016) reported that middle- and upper-income people were more likely to be sedentary because of work, while middle- and lower-income people were more likely to be sedentary because of TV watching or mobile phone use, which may lead to longer periods of sedentary time and more unhealthy snacking. Mielke et al. (2017) noted that physical inactivity among young people is very common worldwide; 81% of young people do not meet the WHO recommendation of at least 1 h of PA per day and that there is some correlation with the level of development; for example, this is true for 79% of young people in LMICs, 84% in high- and middle-income countries, and 79% in high-income countries. Muzenda et al. (2022a) compared the differences between sitting and health risks in high- and middle-income countries and found that when individuals sat for more than 8 h/day, the risk of all-cause and cardiovascular death increased by 29% in low- and middle-income countries compared with 8% in high- and middle-income countries. The authors speculate that this may be because income levels lead to differences in sedentary patterns and their effects (Milton, Macniven & Bauman, 2014). In addition, people in middle- and high-income countries exercise more, while people in low- and middle-income countries exercise less. The higher people’s income is, the more attention they pay to exercise and a healthy lifestyle; this has become an internationally recognized phenomenon (Barbosa Filho et al., 2016). Using physical education interventions in LMICs as a starting point, Ashdown-Franks et al. (2019a) argue that most physical education classes in LMICs do not meet the MVPA recommendations (≥50% MVPA) and that students from low-income minority communities participate in even less PA in school. Black and Asian children are less likely than other children to perform sufficient PA (Vancampfort et al., 2018).

The Global School Student Health Survey (GSHS) is administered to young people in many countries around the world in an effort to develop better health programs (Dema et al., 2019; Page et al., 2013). The GSHS collects basic information about individuals, such as grade, gender, and height (Motamed-Gorji et al., 2019). In addition, the survey includes young people’s recent living habits and obtains information about whether students ate breakfast and other habits for the past 30 days to avoid inaccurate data due to broad answers (Choo, 2013). Drug intervention and sedentary behavior were part of the larger profile, including the frequency of alcohol consumption, drug exposure, and screen time (Long et al., 2021). In addition, Campisi et al. (2020) highlighted this survey because it captures teens’ social relationships, such as whether they are bullied or get into fights. The contribution of the GSHS is that, on the one hand, it evaluates whether the physical condition of teenagers follows the growth law and makes timely suggestions for prevention (World Health Organization, 2017); on the other hand, given the dynamic and unstable situation of teenagers, regular health surveys can improve their psychological quality (Pengpid & Peltzer, 2019). Hoogstoel et al. (2021) concluded that the GSHS promotes sustainable and healthy habits among adolescents and to strengthen students’ ability to cope with social relationships and setbacks. Similarly, Arat & Wong (2016) proposed that participation in the GSHS could subjectively improve the health awareness of adolescents and that the provision of a health index could help students understand methods for evaluating health, thus improving the effectiveness of active intervention. Since the GSHS is conducted on a global scale and has a large amount of data, it is helpful to assess the factors that affect adolescents’ health habits from different perspectives, such as the region and type of school, to propose more targeted suggestions for families, schools and governments (Noel, 2019).

Compared to high-income countries, adolescents in LMICs are typically in the early stages of understanding physical activity and often display low levels of physical activity. Additionally, according to the current literature, there is limited evidence linking PA and SB among school-going adolescents (13–19 years) from LMICs. To fill this research gap, this study primarily aims to synthesize the evidence about physical activity and sedentary behavior in school-going adolescents from LMICs based on the GSHS. It can be hypothesized that youth in low- or middle-income countries would have low levels of physical activity and high levels of sedentary behavior.

## Methods

This systematic scoping review adhered to the methodological frameworks developed by Arksey & O’Malley (2005), Daudt, van Mossel & Scott (2013), Levac, Colquhoun & O’Brien (2010) and the Preferred Reporting Items for Systematic Reviews and Meta-analysis (PRISMA) extension for scoping reviews (Tricco et al., 2018). Scoping review plays a pivotal role in providing a comprehensive overview of a specific research topic, enabling the mapping of the existing literature, clarification of key concepts, identification of research gaps, and outlining future research directions (Arksey & O’Malley, 2005).

### Literature search and identification

**Figure 1:**
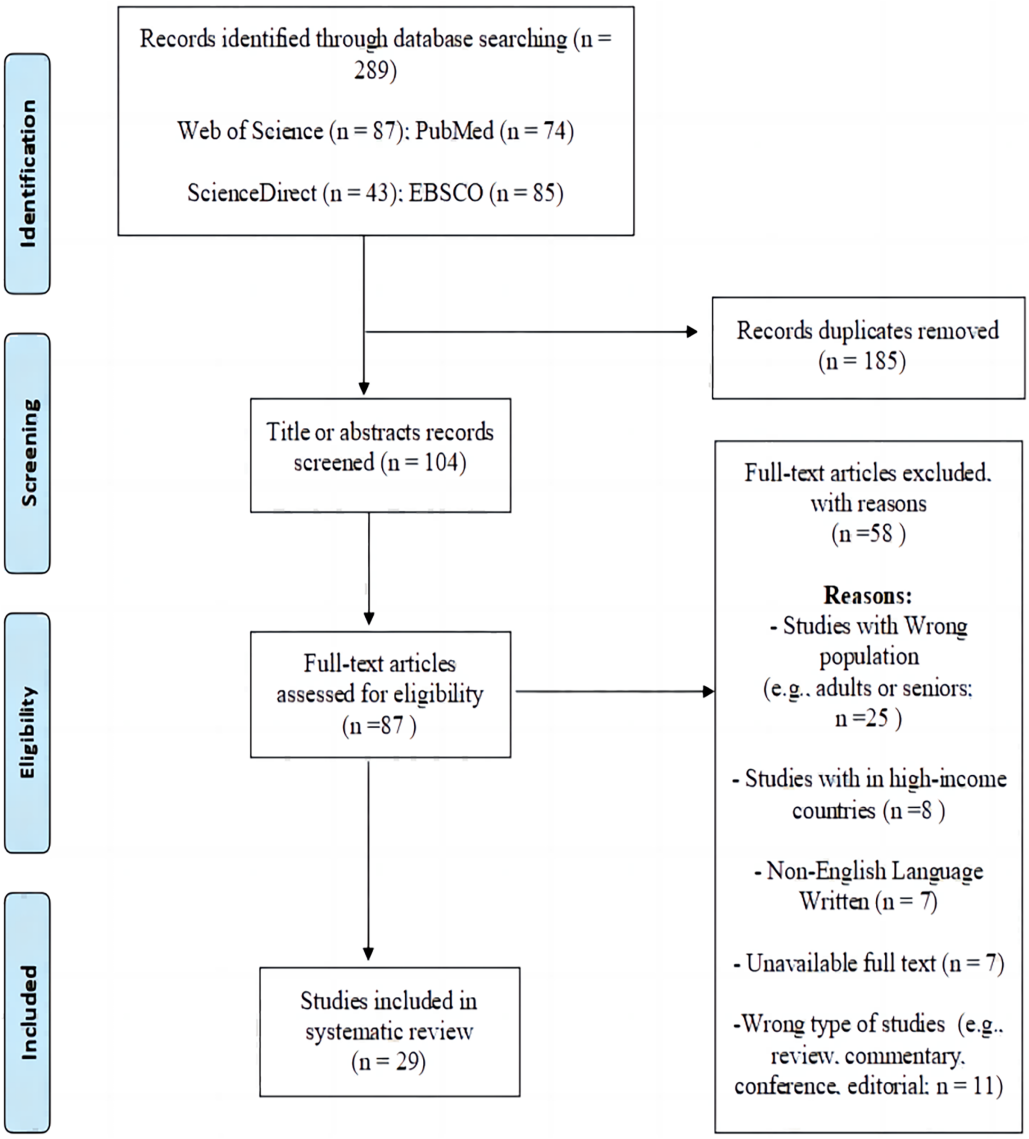
PRISMA flow diagram.

A systematic literature search was conducted across four electronic databases (Web of Science, PubMed, ScienceDirect, EBSCO) on 18^th^ March 2023. The search strategy, outlined comprehensively in Additional File 1, encompasses keywords such as “physical activity”, “sedentary behavior”, “adolescents”, “low- and middle-income countries”, and “the Global Student-based Health Survey”. Following this, the title and abstract, as well as full-text documents, were independently examined by two authors using Endnote software (HL and WZ). The process of literature identification is visually represented in Fig. 1. In the case of discrepancies, a resolution was achieved through consultation with the third author (J Y).

### Eligibility criteria

Inclusion criteria for studies encompassed the following:

1. utilization of data sourced from the Global Student-based Health Survey;
2. exploration of physical activity (defined as any bodily movements generated by skeletal muscle leading to energy expenditure (Caspersen, Powell & Christenson, 1985) and/or sedentary behavior (comprising any waking activities involving standing or sitting that results in an energy expenditure of 1.5 metabolic equivalents (METs) or less during daily life (Bao et al., 2020); (3) specific focus on adolescents;
3. conducting in low- and middle-income countries;
4. study design encompassing observational (*e.g*., cross-sectional, longitudinal) and experimental (*e.g*., RCT and non-RCT controlled trials) studies;
5. published as English journal articles.

The following types of studies were deemed ineligible:

1. those not focused on adolescents, research conducted in high-income countries, studies not utilising data from the Global Student-based Health;
2. non-original studies such as review, commentary, conference, editorial.

### Data extraction and synthesis

In this study, data from each included study was independently extracted by two authors (HL and WZ), using a standardized extraction form within Microsoft Excel. In instances where these two researchers couldn’t reach an agreement on the risk bias assessment, they sought the input of a third assessor (JY) to attain a majority consensus. Before the extraction process commenced, a consensus on the extracted strategy was reached by the authors. The initial phase involved categorizing the included studies based on three research aims, such as correlates, prevalence, and health outcomes of physical activity and/or sedentary behaviour. Subsequently, studies with the same research aim (*e.g*., correlates, prevalence, health outcomes) were extracted and summarised. The specific details obtained from each included study encompassed essential aspects such as first author name, publication year, sample size, study objectives, primary results, and key findings. Additionally, correlates include demographic, socioeconomic status, and environmental factors associated with physical activity and/or sedentary behaviour. We also extracted the data including levels of physical activity such as the percentage of physical activity, physical inactivity, adherence to PA guidelines meetings, active school travel, physical education, and/or sedentary behaviour (*e.g*., hours in sedentary behaviour). Health outcomes such as physiological indicators, psychological indicators, and dietary indicators related to physical activity and/or sedentary behaviour.

## Results

### Overview of studies

The study selection process, along with all the exclusion criteria, is depicted in Fig. 1. The initial search across the four databases yielded 289 articles. After eliminating 185 duplicate articles, 104 articles remained. During the review of paper titles and abstracts, 17 studies were excluded as they did not meet one or more of the inclusion criteria. There was a 98% agreement rate for inclusion/exclusion between the two reviewers (*n* = 2 articles, 2%), with all discrepancies jointly discussed until a decision was made to include or exclude the publication. A thorough assessment of 87 full-text articles was conducted, resulting in the exclusion of 58 articles for various reasons (as shown in Fig. 1). As a result, a total of 29 studies were included in the review (100% agreement).

### Study characteristics and participants

Studies from 2014 to 2016 focused on macro studies on factors and differences affecting physical activity (Table 1). For example, Manyanga et al. (2014) had a sample size of seven adolescents in the United States. The aim is to discover the link between adolescents’ personal habits and physical activity through a broad volume of data. The 2017–2019 study compared youth sports performance in countries with different incomes. Ashdown-Franks et al. (2019b) studied 41 low- and middle-income countries, while Vancampfort et al. (2019a) focused on 52 low- and middle-income countries, with a sample size of more than 150,000. In addition, Ma et al. (2020) correlate the consumption behavior and sports performance of 180,298 adolescents aged 12–15 from middle and low income, to assess the impact of economic background on sports activities and specific performance. Studies after 2020 have focused more on psychological changes in adolescents, and the factor has also been linked to the impact of physical activity. Liu et al. (2021) focused on a sample of 51,702 adolescents with reported depressive symptoms and Ma et al. (2022), a sample of more than 140,000 adolescents with suicidal motives, to confirm the intrinsic link between emotional feedback and sports participation.

**Table 1:** Study characteristics and participants.

References	Study aim	Independent variables	Dependent variables	Outcomes	Study findings
Manyanga et al. (2014)	To evaluate the prevalence of underweight, overweight and obesity as well as associated risk factors among school going adolescents in seven African countries using cross sectional data from the Global School-based Student Health Survey.	Gender Age Food insecurity Vegetable consumption Consistently active Physical education class No walking/biking to school Soft drink Fast food Sitting 3+ h Parental support index	Underweight Overweight and obesity	There is a significant relationship between having 3+ days of physical education and being obese (OR: 1.51; 95% CI [1.02–2.24]). Not walking/biking to school appears to reduce the odds of being overweight in Morocco (OR = 0.77; 95% CI [0.61–0.97]), while it appears to increase the odds for students in Ghana (OR = 1.28; 95% CI [1.01–1.62]). For being obese, sitting 3+ h a day was now significant (OR = 1.58; 95% CI [1.01–2.48]).	Having 3+ days of physical education and not walking/biking to school, sitting 3+ h/day were risk factors with overweight or obesity among school going adolescents.
Peltzer & Pengpid (2016)	To examine the relationship between self-reported leisure time physical inactivity frequency and sedentary behaviour and lifestyle correlates among school children in the Association of Southeast Asian Nations region.	Sex Physical education Hunger (as proxy for socioeconomic status) BMI Fruits Vegetables Bullied Current smoking cigarettes Current other tobacco use Current alcohol use Close friends Lonely Suicidal ideation School attendance (never miss school) Peer support in school Parental or guardian supervision Parental or guardian connectedness Parental or guardian bonding	Physical inactivity Sedentary behaviour	The prevalence of physical inactivity was 80.4%, ranging from 74.8% in Myanmar to 90.7% in Cambodia and sedentary behaviour 33.0%, ranging from 10.5% in Cambodia and Myanmar to 42.7% in Malaysia. 2. Not walking or biking to school, not attending physical education classes, inadequate vegetable consumption and lack of protective factors (peer and parental or guardian support) were associated with physical inactivity. 3. Older age (14 and 15 years old), coming from an upper middle income country, being overweight or obese, attending physical education classes, alcohol use, loneliness, peer support and lack of parental or guardian supervision were associated with sedentary behaviour. 4. In boys, lower socioeconomic status (in the form of having experienced hunger) and coming from a low income or lower middle income country were additionally associated with physical inactivity, and in girls, higher socioeconomic status, not walking or biking to school and being bullied were additionally associated with sedentary behaviour.	(1) The prevalence of leisure physical inactivity and sedentary behaviour among school going adolescents in Association of Southeast Asian Nations countries is very high. (2) Several socio-demographic indicators, health risk status and behaviour, poor mental health and protective factors were identified.
Peltzer & Pengpid (2017)	To assess suicidal ideation and associated factors in school-going adolescents in the Association of Southeast Asian Nations member states.	Socio-demographics (Gender, Age) Hunger Psychosocial distress (Close friends, Lonely, Bullied, Physically attacked, In a physical fight) Parental/guardian support index (Parental or guardian supervision, Connectedness, Bonding) Peer support Current smoking cigarettes Current other tobacco use Ever got drunk Truancy Physical activity Sedentary leisure time behaviour Body Mass Index Country income	Suicidal ideation	No significant association was found between physical activity, sedentary leisure time behaviour and suicidal ideation, While significant associations were found between sedentary leisure time behaviour and suicidal ideation in Malaysia.	There are no significant association was found between physical activity, sedentary leisure time behaviour and suicidal ideation in Association of Southeast Asian Nations countries except Malaysia.
Darfour-Oduro et al. (2018)	To compare fruits and vegetables consumption and physical activity behavior patterns among adolescent girls and boys in 49 low-and-middle-income countries.	Gender	Fruits Vegetables Physical activity	Less than 30% of adolescents across all countries met the World Health Organization guidelines for physical activity. India (29.5%) however had the highest percentage of adolescents meeting recommendations for physical activity. Adolescent boys were more active than girls, and this difference was more notable in the Middle East and North African region.	Adolescents achieving the World Health Organization recommendations for daily consumption of physical activity were consistently low in all countries, and boys were more active than girls,
Vancampfort et al. (2018)	To explore the association between sedentary behavior and depressive symptoms in adolescents from 30 low-and-middle-income countries, controlling for confounders including physical activity.	Sedentary behavior	Depressive symptoms	The prevalence of ≥3 h/day of sedentary behavior was 30.6%. There was a linear increase in the prevalence of depressive symptoms with increasing sedentary time beyond ≥3 h/day (*vs*. <1 h/day). Among boys, 1–2 h/day of sedentary behavior was associated with lower odds for depression (*vs*. <1 h/day). Country wide meta-analysis demonstrated that spending ≥3 h/day *vs*. <3 h/day was associated with a 20% increased odds for depressive symptoms (OR = 1.20; 95% CI [1.16–1.24]) with low between-country heterogeneity (I2 = 27.6%).	Being sedentary for ≥3 h/day is associated with increased odds for depressive symptoms in adolescence.
Ashdown-Franks et al. (2019a)	To examine the association between physical activity and cannabis use among adolescents in 21 low-and-middle-income countries using data from the 2010–2016 Global School-based Student Health Survey.	Cannabis use	Physical activity	The prevalence of adequate levels of physical activity in past and current cannabis use was 7.3% and 6.9%, respectively. Current and past cannabis use (*vs*. never) were associated with a significant 0.62 (95% CI [0.41–0.94]) and 0.43 (95% CI [0.30–0.63]) times lower odds for achieving adequate levels of physical activity, respectively.	(1) High prevalence of low physical activity among adolescents in low-and-middle-income countries. (2) A negative association between cannabis use and adequate physical activity in low-and-middle-income countries.
Ashdown-Franks et al. (2019b)	To examine the relationship between sedentary behavior, fast food consumption and carbonated soft drink consumption among adolescents in 41 low-and-middle-income countries.	Sedentary behavior	Fast food consumption Carbonated soft drink consumption	27.0% of the adolescents engaged in ≥3 h/day of sedentary behavior per day (range: 8.2% (pakistan) to 54.6% (Antigua & Barbuda)). The overall prevalence of sedentary behavior were: <1 h/day 38.7%; 1–2 h/day 34.3%; 3–4 h/day 15.8%; 5–8 h/day 7.7%; and >8 h/day 3.6%. The overall pooled estimates based on a meta-analysis with random effects for the association of ≥3 h/day of sedentary behavior with fast food consumption and soft drink consumption using country-wise estimates were OR = 1.35 (95% CI [1.27–1.43], I2 = 62.1%).) and OR = 1.26 (95% CI [1.19–1.34]; I2 = 54.3%), respectively. Spending >8 h/day of sedentary behavior compared to <1 h/day in females was associated with significantly higher odds for fast food (OR = 1.61, 95% CI [1.38–1.88]) and soft drink consumption (OR = 1.91, 95% CI [1.60–2.28]).	(1) The results demonstrate that among adolescents in low-and-middle-income countries, rates of fast-food consumption and soft drink consumption increased with increasing time spent sedentary. (2) There were some differences in the findings between countries and sexes.
Ashdown-Franks et al. (2019c)	To explore the associations between sedentary behavior and obesity in adolescents from 41 low-and-middle-income countries.	Sedentary behavior	Obesity	The multivariable logistic regression analysis in which >8 h/d of sedentary behavior was associated with 1.40 (95% CI [1.06–1.86]) times higher odds of obesity compared with <1 h/d . The association was strongest in low-income countries (pooled OR = 1.87; 95% CI [1.08–3.25]), followed by lower-middle-income countries (OR = 1.28; 95% CI [1.17–1.41]) and upper-middle-income countries (OR = 1.08; 95% CI [0.99–1.18]).	Being sedentary for ≥ 3 h/d is associated with increased odds of obesity in adolescence.
Vancampfort et al. (2019a)	To explore associations between leisure-time sedentary behavior and loneliness in adolescents from 52 low- and middle-income countries.	Leisure-time sedentary behavior	Loneliness	The prevalence of <1, 1–2, 3–4, 5–8, and >8 h of leisure-time sedentary behavior were 41.4%, 32.9%, 14.8%, 7.4%, and 3.6%, respectively. Compared to those who engage in less than 1 h of leisure-time sedentary behavior per day, the OR (95% CI) of loneliness for 1–2 h/day, 3–4 h/day, 5–8 h/day and >8 h/day were 1.00 [0.91–1.11], 1.29 [1.15–1.45], 1.37 [1.17–1.61], and 1.66 [1.39–1.99], respectively.	Data suggest that leisure-time sedentary behavior is associated with increased odds for feeling lonely in adolescence.
Vancampfort et al. (2019b)	To explore associations between cannabis use and leisure-time sedentary behavior in adolescents from 24 low- and middle-income countries.	Cannabis use	Leisure-time sedentary behavior	The prevalence of high leisure-time sedentary behavior (3 h/day) was 26.6%. Compared to those who did not consume cannabis in the past 30 days, the OR (99% CI) for high leisure-time sedentary behavior among those who used cannabis 1–2 times, 3–9 times, 10–19 times, and 20 times were 0.89 [0.58–1.35], 1.96 [1.26–3.07], 1.97 [0.71–5.47], and 2.34 [0.95–5.78], respectively (test for trend *p* < 0.01).	Data suggest that frequent cannabis use is associated with increased odds for being more sedentary in adolescence.
Vancampfort et al. (2019c)	To assesse at the personal and health-behavior related correlates and at the interpersonal level of the socio-ecological model, correlates of leisure time sedentary behaviour among adolescents aged 12–15 years who participated in the Global school-based Student Health Survey.	Socio-demographic (Age, Sex, Food insecurity) Socio-cultural (Parental support/monitoring, Close friends, Bullying victimization) Other health risk behaviors (Smoking, Alcohol consumption)	Leisure-time sedentary behavior	The overall prevalence of ≥3 h/day of leisure-time sedentary behavior was 26.4% (95% CI [25.6–27.2%]). Increasing age (OR = 1.14; 95% CI [1.11–1.17]), past 30-day smoking (OR = 1.85; 95% CI [1.69–2.03]), alcohol consumption (OR = 2.01; 95% CI [1.85–2.18]), and bullying victimization (OR = 1.39; 95% CI [1.31–1.48]) were positively associated with increased leisure-time sedentary behavior across the entire sample of 181,793 adolescents. Food insecurity (OR = 0.93; 95% CI [0.89–0.97]) and low parental support/monitoring (OR = 0.91; 95% CI [0.85–0.98]) were negatively associated with leisure-time sedentary behavior.	Data indicate that in adolescents aged 12 to 15 years living in low- and middle-income countries, sedentary behavior determined by modifiable sociocultural and lifestyle factors (age, past 30-day smoking, alcohol consumption, bullying victimization, food insecurity and parental support/monitoring).
Vancampfort et al. (2019b)	To identifie physical activity correlates using data from the Global school-based Student Health Survey.	Age Sex Food insecurity Smoking Alcohol consumption Fast food consumption Carbonated soft drink consumption Low fruit/vegetable consumption Obesity Physical education Low parental support/monitoring Close friends Bullying victimization	Physical activity	The prevalence of low physical activity was 15.3% (95% CI [14.5–16.1%]). Boys (OR = 1.64; 95% CI [1.47–1.83]) and those who participated in physical education for ≥5 days/week (OR = 1.12; 95% CI [1.10–1.15]) were more likely to meet physical activity guidelines, while adolescents with food insecurity (OR = 0.85; 95% CI [0.80–0.90]), low fruit and vegetable intake (OR = 0.68; 95% CI [0.63–0.74]), low parental support/monitoring (OR = 0.68; 95% CI [0.62–0.74]), no friends (OR = 0.80; 95% CI [0.72–0.88]), and who experienced bullying (OR = 0.93; 95% CI [0.86–0.99]) were less likely to have adequate levels of physical activity.	Data indicate that in adolescents aged 12 to 15 years living in low- and middle-income countries, physical activity determined by modifiable sociocultural and lifestyle factors (sex, physical education, food insecurity, fruit and vegetable intake, parental support/monitoring, friends, and bullying victimization).
Alfonso-Rosa et al. (2020)	To provide a global perspective of the association between different lifestyle behaviors and bullying in school adolescents and to ascertain whether or not the Human Development Index moderated those associations.	Lifestyle behaviors: Physical activity Active transport Physical education class participation Sedentary behavior	Bullying	Excessive sitting time [1.38 (1.34, 1.41)], attendance to physical education [0.87 (0.85, 0.89)], and active transport [0.94 (0.91, 0.97)] but not overall physical activity [1.01 (0.99, 1.04)] were associated with bullying in the study sample.	Active commuting and attendance to physical education play a protective role for bullying, while physical activity does not.
Darfour-Oduro, Andrade & Grigsby-Toussaint (2020)	To determine the relationship between the physical and social environments as well as physical activity on the daily intake of fruits and vegetables among adolescents in low- and middle-income countries.	Food insecurity Parental connectedness Parental Supervision Parental Bonding Physical Activity	Fruits Vegetables	Physical activity was also positively associated with adolescents consuming ≥5 servings of fruits and vegetables daily (AOR = 1.30; 95% CI [1.13–1.50]; *p*-value < 0.001).	Adolescents’ physical activity behavior is a significant predictor of adolescents’ intake of fruits and vegetables.
Li et al. (2020)	To compare the prevalence of fast-food consumption among young adolescents in low- and middle-income countries.	Age Sex Body Mass Index Food insecurity Fruit consumption Vegetable consumption Soft-drink consumption Smoking Physical active Sedentary behavior.	Fast-food consumption	Physical activity, and sedentary behavior [odds ratio: 1.258 (1.209–1.308), and 1.491 (1.439–1.545)] were positively correlated with fast food consumption.	Physical activity, and sedentary behavior were positively correlated with fast food consumption.
Liu et al. (2021)	This study aimed to explore the association between active school travel and depressive symptoms among adolescents aged 12-15 yeas from 26 low- and middle-income countries.	Active school travel	Depressive symptoms	The prevalence of active school travel were 37.0%. Compared with those not having active school travel, adolescents with active school travel were less likely to have self-reported depressive symptoms (OR = 0.88, 95% CI [0.85–0.93]) regardless of gender. Countrywide meta-analysis demonstrated that having active school travel *vs*. not having active school travel was associated with 12% decreased odds for depressive symptoms (OR = 0.88; 95% CI [0.82–0.94]) but with a moderate between-country heterogeneity (I2 = 59.0%)	AST may be an effective prevention against depressive symptoms among adolescents from low- and middle-income countries.
Ma et al. (2020)	To assesse the prevalence of physical activity and sedentary behavior, and their associations with a country’s economic development in young adolescents aged 12–15 years in 68 low- and middle-income countries.	Purchasing power parity/capita	Physical activity Sedentary behavior	15.3% of young adolescents achieved the recommended level for sufficient physical activity (≥60 min/day of physical activity of any kind per week according to World Health Organization) and 64.6% achieved a low sedentary behavior (≤2 h of sitting activities/day according to some guidelines, not accounting for sitting time at school or for doing homework). Only 9.1% of young adolescents met the recommended levels of both behaviors. Comparing the lowest to the highest quintiles of a country’s purchasing power arity *per capita* , mean values of both physical activity (boys: 2.55 to 2.96 days/week; girls: 2.10 to 2.31 days/week) and sedentary behavior (boys: 1.86 to 3.13 h/day; girls: 1.83 to 3.53 h/day) increased. The prevalence of having both recommended behaviors decreased among boys (12.0% to 10.0%) and girls (9.6% to 4.9%) (*p* < 0.001).	(1) A relatively low prevalence of sufficient physical activity and relatively high prevalence of sedentary behavioramongyoung adolescents aged 12–15 years in low- and middle-income countries. (2) Physical activity and sedentary behavior differed according to sex, age, country, and Purchasing power parity/capita.
Uddin et al. (2020)	To examine the relationships of physical activity and sedentary behaviour with suicidal thoughts and behaviour among adolescents in low- and middle-income countries.	Physical activity Sedentary behaviours	Suicidal ideation Suicide planning Suicide attempts	High sedentary behavior (≥3 h/day) was independently associated with higher odds of suicidal ideation, planning, and attempts for both male and female adolescents. Insufficient physical activity (<60 min/day) was not associated with higher odds of ideation for either sex; however, was associated with planning and attempts for male adolescents. The combination of insufficient physical activity and high sedentary behavior, compared to sufficient physical activity and low sedentary behavior, was associated with higher odds of suicide ideation and planning for both male and female adolescents, and suicide attempts for male adolescents	(1) High sedentary behavior may be an indicator of suicidal vulnerability among adolescents in low- and middle-income countries. (2) Low physical activity may be a more important risk for suicidal thoughts and behaviours among male, than female, adolescents.
Wang et al. (2020)	To examine the association between sedentary behaviour and the risk of anxiety symptoms among youth in 24 low- and middleincome countries.	Sedentary behaviour	Anxiety symptoms	Sedentary behaviour of >2 h/day was observed in 32.7% ofthe youth (ranging from 9.6% in Myanmar to 53.5% in Saint Lucia). Countrywide meta-analysis demonstrated that sedentary behaviour of >2 h/day (*vs*. ≤2 h/day) was associated with an increased risk of anxiety symptoms (OR = 1.22; 95% CI [1.10–1.37])	The current study provides multi-national evidence of the dangerous effect of sedentary behaviour on anxiety symptoms among youth in low- and middle-income countries.
Xu et al. (2020)	To describe and comare the separate and combined prevalence of physical activity, active transportation, physical education, and sedentary behavior among adolescents 12–15 year-olds in low- and middle-income countries.	N/A	Physical activity, Active transportation Physical education Sedentary behavior	Only 0.7% (95% confidence interval (CI) [0.5–1.0%]) of the adolescents, comprising 0.9% (95% CI [0.6–1.3%]) of the boys and 0.5% (95% CI [0.3–0.7%]) of the girls, displayed all of the qualifying physical behaviors. The overall prevalence of physical activity, active transportation, physical education, and sedentary behavior was 15.2% (95% CI [13.7–16.7%]), 39.5% (95% CI [34.9–44.0%]), 18.8% (95% CI [16.1–21.5%]), and 34.6% (95% CI [28.4–40.7%]), respectively. The overall prevalence of high levels of combined physical behaviors was 6.6% (95% CI [5.4–7.8%]), with lowest in the Eastern Mediterranean region (4.9%, 95% CI [3.5–6.2%]) and highest in Southeast Asia (8.6%, 95% CI [4.9–12.3%]).	The prevalence of the separate physical behaviors and high levels of the combined physical behaviors was consistently low among young adolescents in low- and middle-income countries, and that of all qualifying physical behaviors was even lower.
Aboagye et al. (2021)	This study seeks to determine the prevalence and factors influencing unintentional injuries among in-school adolescents using nationally representative data.	Sociodemographic Characteristics (Age, Sex, Grade) Hunger (A proxy to socio-economic status) Physical education Truancy Bullied Physical fight Physically attacked peer support substance use (current alcohol use, smoking cigarettes, tobacco use, marijuana use, drugs use) psychological distress (Loneliness, Anxiety, Suicide attempt, Close friends) Parental or guardian support and bonding (Parental supervision, Parental Connectedness, Parental or guardian Bonding, Parental respect for privacy)	Self-reported injury	Over sixty-six percent of the students in junior high schools reported having been injured, while 63.1% of students aged 14–16 years were injured. Among those who reported having been injured once or multiple times, hunger, physical education, truancy, physical fighting, physical attack, and peer support contributed to 63.9%, 73.8%, 67.9%, 72.8%, 75.9%, 75.4%, and 53.4% of the prevalence, respectively. In-school adolescents who participated in physical education (adjusted OR = 1.27, CI [1.03–1.58]) had higher odds of reporting one or more serious injuries.	In-school adolescents who participated in physical education had higher odds of reporting one or more serious injuries.
Hoogstoel et al. (2021)	To assess the prevalence of suicidality, identify the different behavioral profiles related to lifestyle, and determine their association with suicidality among adolescents in Benin using latent class analysis.	Psychosocial distress (Anxiety, Loneliness, Physical attack, Physical fight) Socio-environmental factors (parental support) Health Risk Behaviors (Tobacco consumption, Alcohol consumption, Physical activity) Sociodemographic characteristics (Age, Sex, Grade, Socioeconomic status)	Suicidal ideation Suicidal planning Suicidal attempts	A total of 62.0% had insufficient physical activity (inactive). Physical inactivity and a lack of parental support were relatively common indicators in each profile. However, it should be noted that the adolescents in Profiles 2 and 4 were much more active than those in Profiles 1 and 3. A lack of parental support was a very prominent variable in each profile, with a slight gradual increase in the proportions across the profiles from 1 to 4; Profile 4 had the most adolescents in need of support.	This increased risk of suicidality was associated with physical inactivity.
Ozeylem, de la Torre-Luque & Essau (2021)	To examine personal and interpersonal factors that contribute to risk for substance use among adolescents in six Association of Southeast Asian Nations low- and middle-income countries (*i.e*., Indonesia, Laos, Malaysia, Myanmar, Philippines and Thailand).	Personal risk factors: Sex Age The frequency of being physically active Have you been so worried about something that you could not sleep at night Interpersonal risk factors: Being bullied, Loneliness Having close friends Feelings that your parents or guardians understand their problems and worries.	Alcohol use Problematic alcohol use Regular smoking Other drugs	The prevalence of physical activity practice (4 days a week or more) was 19.4% (Indonesia), 23.7% (Laos), 30.18% (Malaysia), 21.66% (Myanmar), 15.2% (Philippines), 24.71% (Thailand), respectively. Comphysical activityred with those who hadless than 4 days/week of physical activity practice, those who had 4 days a week of physical activity practice were at higher odds ratio of Binge drinking OR = 1.33 (95% confidence interval (CI) [1.15–1.54]), Heavy drinking OR = 2.00 (95% CI [1.50–2.67]).	Regular physical activity were found to predict adolescents heavy and binge drinking.
Qi et al. (2021)	To estimate the prevalence of respondents reporting a poor, intermediate, and ideal smoking status, body mass index, physical activity level, and healthy dietary pattern, as well as the cumulative numbers of ideal cardiovascular health behaviours, among young adolescents in low- and middle-income countries by region and country.	N/A	Smoking status Body Mass Index Physical activity level Healthy dietary pattern	Regarding physical activity, the overall prevalence of an ideal level was 15.4% (13.7–17.2), and the lowest and highest national rates were observed in Cambodia and Bangladesh, respectively. Boys and girls had prevalence rates of 19.7% (17.8–21.6) and 11.5% (10.0–13.0), respectively. Boys were more likely than girls to report an ideal level of physical activity (*P* = 0.001). The pooled prevalence of an ideal level of physical activity was lowest in the Eastern Mediterranean (12.9%) and highest in Southeast Asia (18.8%).	Consistently low proportions of young adolescents in low- and middle-income countriess met the ideal levels of physical activity.
Smith et al. (2021)	To assess the associations between obesogenic behaviours and bullying victimization in 54 low- and middle-income countries.	Anxiety-induced sleep problems Fast-food consumption Carbonated soft-drink consumption No physical activity Sedentary behaviour	Bullying victimization	A total of 31.4% (no physical activity); 26.4% (≥3 h/day sedentary behaviour) Bullying victimization (*vs* no bullying victimization) was significant associated with no physical activity 0.84 (0.79–0.89); and sedentary behaviour 1.34 (1.25–1.43). The strength of the association was similar among boys and girls.	Bullying victimization was significant associated with no physical activity and sedentary behaviour.
Vancampfort et al. (2021)	To examine associations between leisure-time sedentary behavior and moderate-to-vigorous physical activity in adolescents from 47 low- and middle-income countries .	Leisure-time sedentary behavior	Moderate-to-vigorous physical activity	The prevalence of ≥3 h/day of leisure-time sedentary behavior and 60 min of moderate-to-vigorous physical activity/day last week were 26.3% (girls 26.2%; boys 26.5%) and 15.3% (girls 12.1%; boys 18.4%), respectively. Leisure-time sedentary behavior of ≥3 h/day *vs*. <3 h/day was associated with a 35% increased odds for adequate levels of moderate-to-vigorous physical activity in boys [OR ¼ 1.35 (95% CI ¼ [1.23–1.48]) and 22% in girls (1.22 (95% CI ¼ [1.10–1.36]).	Data indicate that being physically active 60 min per day every day and at moderate-tovigorous intensity and being sedentary ≥3 h/day during leisure-time might co-exist in adolescents in some low- and middle-income countries.
Khan, Khan & Burton (2022)	To evaluate the associations of physical activity and sedentary behaviour with loneliness among adolescents with overweight/obesity.	Physical activity Sedentary behaviour	Loneliness	A total of 31% had high sedentary behavior (3 h/day) and 86% were not sufficiently active (<7 days/week of 60 min/day). High sedentary behavior and insufficient physical activity were positively associated with loneliness (a OR 1.22, 95% CI [1.08–1.38], a OR 1.37, 95% CI [1.18–1.59], respectively).	High sedentary behavior and insufficient physical activity were positively associated with loneliness.
Ma et al. (2022)	To examine the association between leisure sedentary time and suicidal ideation, planning and attempts among adolescents in low- and middle-income countries.	Sedentary behaviour	Suicidal ideation Suicidal planning Suicidal attempts	The overall prevalence of leisure sedentary time more than 2 h/day was 35.3%, lowest in Nepal (9.5%) and highest in Kuwait (63.5%). Compared with those who had less than 1 h/day of sedentary time, those who had 3, 4 h/day sedentary time were at higher odds ratio (OR) of suicidal ideation OR = 1.21 (95% confidence interval (CI) [1.14–1.29]), planning OR = 1.15 (95% CI [1.07–1.22]) and attempts OR = 1.17 (95% CI [1.09–1.26]), and those who had more than 8 h/day sedentary time were at OR = 1.58 (95% CI [1.44–1.72]), OR = 1.44 (95% CI [1.31–1.58]) and OR = 1.27 (95% CI [1.16–1.40]), respectively.	Higher amounts of leisure sedentary time are associated with suicidal ideation, planning and attempts among adolescents.
Felez-Nobrega et al. (2023)	The present study aims to examine temporal trends in adolescents’ active school commuting and to examine if there are diferences in such trends by sex.	Active commuting	Sex (male or female)	Trends in active school commuting were heterogeneous across countries, with results showing stable patterns for the majority (16/28), decreasing trends for some (7/28) and increasing trends over time for a few (5/28). The majority of countries showed no differences in active school commuting trends between girls and boys.	The quantification of changes in adolescents’ active school commuting over time, together with a a deeper understanding of local determinants for such behaviors will provide valuable evidence to inform the development of tailored and context-specific actions

### Levels of physical activity or sedentary behavior

Most studies have reported a high level of sedentary behavior and low-level physical activity in low- and middle-income countries (Table 2). Peltzer & Pengpid (2016) confirmed this by looking at the rates of sedentary and physical activity participation in different countries: 42.7% of youth in Malaysia were associated with a higher probability of physical inactivity (90.7%), compared with 74.8 % of youth in Cambodia. Xu et al. (2020) reflected that the overall prevalence of high levels of combined physical behaviours was 6.6% (95% CI [5.4–7.8%]), with the lowest in the Eastern Mediterranean region (4.9%, 95% CI [3.5–6.2%]) and highest in Southeast Asia (8.6%, 95% CI [4.9–12.3%]). However, Vancampfort et al. (2019b) and other scholars proposed that there was no significant difference in the 3 h/day of leisure time sedentary behavior between boys and girls (girls 26.2%; boys 26.5%), while there was a difference of 6% between boys and girls in physical activity participation, suggesting that sedentary behavior has less interference with physical activity participation. Similarly, some scholars have found large differences in physical participation across countries, but not in the probability of sedentary behavior (Ozeylem, de la Torre-Luque & Essau, 2021; Qi et al., 2021).

**Table 2:** Levels of physical activity or sedentary behaviour.

	PA	SB
Caleyachetty et al. (2015)	71.4% (69.5–73.3) reported low physical activity	N/A
Peltzer & Pengpid (2016)	The prevalence of physical inactivity was 80.4%, ranging from 74.8% in Myanmar to 90.7% in Cambodia	Sedentary behaviour 33.0%, ranging from 10.5% in Cambodia and Myanmar to 42.7% in Malaysia.
Darfour-Oduro et al. (2018)	Adolescents in India were the most active (29.5%) in terms of meeting the WHO recommendation for daily PA	N/A
Vancampfort et al. (2018)	N/A	The prevalence of ≥3 h/day of sedentary behaviour were 30.6%
Ashdown-Franks et al. (2019a)	The prevalence of adequate levels of PA in the past was 7.3%.	N/A
Ashdown-Franks et al. (2019b)	N/A	The overall prevalence of SB were: <1 h/day 38.7%; 1–2 h/day 34.3%; 3–4 h/day 15.8%; 5–8 h/day 7.7%; and > 8 h/day 3.6%.
Ashdown-Franks et al. (2019c)	N/A	The prevalence of SB was as follows: <1 h/d: 38.4%; 1 to 2 h/d: 35.4%; 3 to 4 h/d: 15.6%; 5 to 8 h/d: 7.3%; and >8 h/d: 3.4%.
Vancampfort et al. (2019a)	N/A	The prevalence of <1, 1–2, 3–4, 5–8, and >8 h of leisure-time sedentary behaviour were 41.4%, 32.9%, 14.8%, 7.4%, and 3.6%.
Vancampfort et al. (2019b)	N/A	The prevalence of high leisure-time sedentary behaviour was 26.6% while 2.8% used cannabis at least once in the past 30 days (1–2 times 1.4%; 3–9 times 0.8%; 10–19 times 0.3%; ≥20 times 0.3%).
Vancampfort et al. (2019c)	N/A	The overall prevalence of ≥3 h/day of leisure-time sedentary behaviour was 26.4% (95% CI [25.6–27.2%]).
Vancampfort et al. (2019b)	The overall prevalence of adequate physical activity was 15.3% (95% CI [14.5–16.1%])	N/A
Liu et al. (2021)	The prevalence of active school travel was 37.0%	N/A
Ma et al. (2020)	A total of 15.3% of young adolescents achieved the recommended level for sufficient physical activity	A total of 15.3% of young adolescents achieved and 64.6% achieved a low sedentary behaviour
Wang et al. (2020)	N/A	Sedentary behaviour of >2 h/day was observed in 32.7% of the youth (ranging from 9.6% in Myanmar to 53.5% in Saint Lucia)
Xu et al. (2020)	The overall prevalence of physical activity, active transportation, physical education, and sedentary behaviour was 15.2% (95% CI [13.7–16.7%]), 39.5% (95% CI [34.9–44.0%]), and 18.8% (95% CI [16.1–21.5%]).	The overall prevalence of sedentary behaviour was 34.6% (95% CI [28.4–40.7%]).
Aboagye et al. (2021)	Among those who reported physical education contributed to 67.9%, of the prevalence.	N/A
Hoogstoel et al. (2021)	A total of 62.0% of the adolescents had insufficient physical activity (inactive).	N/A
Ozeylem, de la Torre-Luque & Essau (2021)	The percentage of physical activity practice in Indonesia, Laos, Malaysia, Myanmar, Philippines and Thailand was 19.4%, 23.7%, 30.18%, 21.66%, 15.2%, and 24.71%.	N/A
Qi et al. (2021)	Overall, 15.4% (13.7–17.2) of respondents reported the ideal levels for physical activity	N/A
Smith et al. (2021)	The prevalence of 31.4% (no physical activity)	The prevalence of 26.4% (≥3 h/day sedentary behaviour).
Vancampfort et al. (2021)	The prevalence of MVPA/day last week were 15.3% (girls 12.1%; boys 18.4%)	The prevalence of 3 h/day of leisure-time sedentary behaviour were 26.3% (girls 26.2%; boys 26.5%)
Khan, Khan & Burton (2022)	Overall, 86% were not sufficiently active (<7 days/week of ≥60 min/day).	Overall, 31% had high SB (≥3 h/day)
Ma et al. (2022)	N/A	The overall prevalence of leisure sedentary time more than 2 h/day was 35.3%, lowest in Nepal (9.5%) and highest in Kuwait (63.5%).

### Benefits of physical activity or sedentary behavior

Manyanga et al. (2014) have analyzed that physical education is an effective measure for obesity intervention, and the relationship between effective sports participation and obesity intervention presents a positive relationship (OR: 1.51; 95% CI [1.02–2.24]) (Table 3). In addition, Darfour-Oduro, Andrade & Grigsby-Toussaint (2020) also confirmed that physical activity may play a role in increasing the intake of vegetables and fruits or decreasing the consumption of fast food in adolescents, which also feeds back into effective obesity interventions (AOR= 1.30; 95% CI [1–13-1.50]; *p*-value < 0.001). From the perspective of suicidal ideation, there was a meaningful positive association between insufficient PA and suicidal ideation for males in the African region (OR 1.36; 95% CI [1.15, 1.60]) (Uddin et al., 2020). From the perspective of emotion regulation, Liu et al. (2021) analyzed the inverse correlation between physical activity and depressive symptoms, finding that those not having active school travel (AST), and youth with AST were less likely to have self-reported depressive symptoms (OR = 0.88, 95% CI [0.85–0.93]) regardless of gender and Khan also analyzed that insufficient physical activity would increase the probability of loneliness (aOR 1.37, 95% CI [1.18–1.59]). More significantly, higher rates of participation in physical activity corresponded to lower rates of suicide (95 CI [1.14–1.29]) (Ma et al., 2022).

**Table 3:** Benefits of physical activity or sedentary behaviour.

	PA	Summary	Physiological indicators	Psychological indicators	Dietary indicators	SB	Summay	Physiological indicators	Psychologicl indicators	Dietary indicats
Manyanga et al. (2014)	Significant relationship between having 3+ days of physical education and being obese (OR: 1.51; 95% CI [1.02–2.24]).	Physical education may be a contributor of obesity prevention	Obesity			No significant relationship between sitting 3+ h a day and being obese (OR = 1.58; 95% CI [1.01–2.48]).	Sedentary behaviour was not associated with obesity			
Peltzer & Pengpid (2017)	No significant associations were found between physical activity and suicidal ideation.	Physical activity was not associated with suicidal ideation				No significant associations were found between sedentary leisure time behavior and suicidal ideation.	Sedentay behavior was not associatd with suicidal ideation			
Vancampfort et al. (2018)	N/A	NA				Compared to <1 h/day of sedentary behaviour for, ≥3 h were associated with significant increasing odds for depressive symptoms.	Sedentay behavior may be a contributor to depressie symptos		Depressive symptoms	
Ashdown-Franks et al. (2019b)	N/A	NA				Fast-food consumption and soft drink consumption increased with increasing time spent sedentary.	Increased fast-food consumption and soft drink consumption may be a contributor to sedentary behavior			Fast-food consumption and soft drink consumption
Ashdown-Franks et al. (2019c)	N/A	NA				SB for ≥3 h/d was associated with higher odds of obesity in 32 countries.	Sedentay behavior for ≥3 h/dmay be a contribute or to obesity	Obesity		
Vancampfort et al. (2019a)	N/A	N/A				Leisure-time sedentary behaviour is associated with increased odds of feeling lonely in adolescence	Leisure-time sedentary behaviour may be a contributor to feeling lonely in adolescence	Leisure-time sedentary behaviour		
Alfonso-Rosa et al. (2020)	Physical activity does not affect adolescents regarding bullying.	Physical activity was not associated with bullying				Excessive sitting time was associated with bullying	Excessive sitting time may be a contributor to bullying	Bullying		
Darfour-Oduro, Andrade & Grigsby-Toussaint (2020)	Physical activity was also positively associated with adolescents’ intake of fruits and vegetables	Physical activity may be a contributor to adolescents’ intake of fruits and vegetables			Intake of fruits and vegetabls	N/A	N/A			
Li et al. (2020)	Physical activity was positively correlated with fast food consumption	Physical activity may be a contributor to the fast food consumption			Fast food consumption	Sedentary behaviour was positively correlated with fast food consumption	Sedentary behaviour may be a contributr to the fast food consumption			Fast food consumption
Liu et al. (2021)	Compared with those not having active school travel, adolescents with active school travel were less likely to have self-reported depressive symptoms	Active school travel may be a contribution to reduce the self-reported depressive symptoms		Depressive symptoms		N/A	N/A			
Uddin et al. (2020)	Insufficient PA was associated with higher odds of suicide ideation and planning for both male and female adolescents, and suicide attempts for male adolescents.	Insufficient PA may be a contributor to higher odds of suicide ideation and planning for both male and female adolescents, and suicide attempts for male adolescents		Suicide ideation		High sedentary behaviour (≥3 h/day) was independently associated with higher odds of suicidal ideation	High sedentary behaviour (≥3 h/day) may be a contributor to suicidal ideation		Suicidal ideation	
Wang et al. (2020)	N/A	N/A				Sedentary behaviour of >2 h/day was associated with an increased risk of anxiety symptoms.	Sedentary behaviour of >2 h/day may be a contributr to an increased risk of anxiety symptoms		Anxiety symptoms	
Aboagye et al. (2021)	In-school adolescents who participated in physical education had higher odds of reporting one or more serious injuries.	Participated in physical education may be a contributor to the higher odds of reporting one or more serious injuries.	Injuries			N/A	N/A			
Hoogstoel et al. (2021)	Physical inactivity and a lack of parental support were relatively common indicators in each profile	Physical inactivity and a lack of parental support may be a contributor to common indicators in each profile	Violence	Psychological distress	Alcohol and tobacco	N/A	N/A			
Ozeylem, de la Torre-Luque & Essau (2021)	Physical activity was positively correlated with binge drinking and heavy drinking.	Physical activity may be a contributor to binge drinking and heavy drinking.				N/A	N/A			
Smith et al. (2021)	Bullying victimization (*vs* no bullying victimization) was significantly associated with greater odds for all types of obesogenic behaviour with the exception of physical activity	Bullying victimization was not associated with physical activity				Bullying victimization (*vs* no bullying victimization) was significantly associated with greater odds for sedentary behaviour, which showed an inverse association.	Bullying victimization may be a contributor to greater odds for sedentary behaviour	Bullying		
Khan, Khan & Burton (2022)	Insufficient PA was positively associated with loneliness.	Insufficiet PA may be a contributor to the loneliness		Loneliness		High SB was positively associated with loneliness.	High sedentary behaviour may be a contributor to loneliness		Loneliness	
Ma et al. (2022)	People who have higher physical activity levels, which were associated with lower suicidal ideation	Higher physical activity levels may be a contributor to the low suicidal ideation		Low suicidal ideation		People who had 3,4 h/day sedentary time was at higher odds ratio (OR) of suicidal ideation	3,4 h/day sedentary time may be a contributor to higher odds ratio (OR) of suicidal ideation		Suicidal ideation	

### Factors of physical activity or sedentary behavior

There are significant differences in both physical participation and sedentary behaviour between boys and girls (Table 4), with higher social status girls having higher rates of sedentary behaviour, while boys from low-income families generally have lower rates of physical activity participation (the prevalence of physical inactivity: 80.4%) (Peltzer & Pengpid, 2016). Similarly, Ma et al. (2020) indicate that the mean duration (h/day) of sedentary behaviour among both sexes was lowest in Southeast Asia (boys: 1.89, 1.38–2.40; girls: 1.82, 1.26–2.37). Families with high consumption levels generally attach more importance to health, which can be directly reflected in the importance of physical activity. In addition, the choice of sports activities in different countries has a great influence, especially the difference in education system makes the difference in the frequency of physical activity of adolescents in different countries of the same age group greater. For example, although the coverage rate of sedentary behaviour is not much different between Middle Eastern countries and North America, it is more common for adolescents in North America to meet the standard of physical activities (*e.g*., Morocco: 29.5% and India: 29.5%) (Darfour-Oduro et al., 2018). This corresponds to Vancampfort et al. (2019b) view that policy guidance can reflect the focus of national development, and thus is an important indicator of youth sports participation.

**Table 4:** Factors of physical activity or sedentary behaviour.

	PA	SB
Peltzer & Pengpid (2016)	In boys, lower socioeconomic status (in the form of having experienced hunger) and coming from a low-income or lower middle-income country.	In girls, higher socioeconomic status, not walking or biking to school and being bullied.
Darfour-Oduro et al. (2018)	Cultural and gender norms from the Middle East and North Africa.	N/A
Ashdown-Franks et al. (2019a)	Unique sociodemographic and environmental characteristics in low- and middle-income countries.	N/A
Vancampfort et al. (2019b)	N/A	Frequent cannabis use
Vancampfort et al. (2019c)	N/A	Socio-demograhic, sociocultural, socio-economic, and health behavior related factors
Vancampfort et al. (2019b)	Sociocultural, socio-economic, and policy-related factors	N/A
Ma et al. (2020)	Sex, WHO Region, and purchasing power parity category	Sex, WHO Region, and purchasing power parity category
Vancampfort et al. (2021)	N/A	Feeling lonely in adolescence

## Discussion

Compared with adolescents in high-income countries, adolescents in LMICs are still in the initial stage of understanding physical activity (*i.e*., a low level of PA) (Owen et al., 2022). Therefore, they are reluctant to spend time on sports activities in venues with professional sports facilities. People in these areas have low educational levels, are exposed to fewer recreational activities, and pay little attention to their health. They prefer to spend their spare time on other recreational activities and are less willing to spend time on PA. Second, transportation in LMICs is often inconvenient, which affects the construction of sports venues and the transportation and installation of sports facilities, resulting in a shortage of sports facilities (Psaki et al., 2022). Due to the low economic level and slow development speed of these countries, the sports equipment cannot meet the needs of children’s sports activities, and new investments in the sports field cannot easily keep up with population growth (Muzenda et al., 2022a). These two factors cause a shortage of sports facilities in low- and middle-income areas. Third, a previous study noted that fewer children participate in physical exercise in LMICs (Snilstveit et al., 2016). Teenagers’ enthusiasm for participating in sports activities is not high because families in LMICs do not pay sufficient attention to their children’s participation in sports (Klasen & Crombag, 2013) or lack understanding and support. Furthermore, many children are afraid to participate or have no time to participate in sports activities, which hinders sports development (Shields & Synnot, 2016). Fourth, insufficient investment in sports funds in LMICs is also an important factor that affects sports development (Psaki et al., 2022). Because the local government has limited funds, it puts more funds into infrastructure construction and improving people’s living conditions, and the small amount of remaining funds can be used to develop sports. A number of studies have shown that private enterprises in LMICs are not willing to invest money in the development of sports (Bettin & Zazzaro, 2018; Giulianotti, Collison & Darnell, 2021; Schulenkorf & Schlenker, 2017) because it does not produce enough economic returns to enterprises in a short time. When private enterprises do not obtain enough profits, their enthusiasm for capital investment is not high. Fifth, LMICs have a lower level of technical skill in sports activities, lower training levels, and fewer sporting events due to limited funds. More importantly, LMICs lack excellent coaches, management, and professional talent, all of which lead to fewer local youth sports activities (Abdel-All et al., 2017; Atkins et al., 2016; Hennessy et al., 2022). Therefore, suggestions for low- and middle-income countries to pay more attention to physical education should first focus on educational system reform. Currently, for example, the main problems in China and India are insufficient educational resources for the population. Although measures cannot be implemented in the short term, some measures, such as the reform of the household registration system or the improvement of the education evaluation system, can be guaranteed through efforts. Another important measure is the continuous improvement of public health awareness because the popularization of basic health knowledge requires the support of the government and the media. This finding corresponds to Muzenda et al. (2022b) suggestion that low- and middle-income groups have a superficial understanding of the purpose and significance of sports and fail to internalize this into rational and conscious behavior. It is necessary to cultivate the awareness that sports participation is linked with personal development and economic improvement.

This study revealed that levels of physical activity and sedentary behavior are important indicators of health problems, including obesity and disease risk. There is a significant difference between PA and SB, with some studies showing a higher proportion (29.5%) of people meeting the World Health Organization’s recommended level of physical activity (Aubert et al., 2021). However, physical activity levels vary significantly between studies and regions, with relatively low rates of physical inactivity in some countries, such as Myanmar (74.8%), and rates as high as 90.7% in Cambodia (Peltzer & Pengpid, 2016). This difference may be related to a variety of factors, such as culture, infrastructure, education level, and socioeconomic factors (Darfour-Oduro et al., 2018). This finding corresponds to Firth et al. (2020) view that urbanization may lead to changes in people’s lifestyles, resulting in lower levels of physical activity. In addition, several studies have provided evidence that age, gender, and family economic status may influence participation in physical activity (Manyanga et al., 2014). Similar differences were observed for the incidence of sedentary behavior. The low prevalence of sedentary behavior in some developing countries (10.5%) and in developed countries (42.7%) may be related to factors such as the composition of the labor force, leisure habits, and modes of transport in different countries (Vancampfort et al., 2019a). This also raises questions about differences in the levels of physical activity and sedentary behavior among countries with different income levels. Liu et al. (2021) suggested that some countries have lower rates of sedentary behavior because they have more labor-intensive industries. In addition, increasing screen time and the use of electronic devices may lead to an increase in sedentary behavior. Darfour-Oduro, Andrade & Grigsby-Toussaint (2020) noted that school sports facilities, family education, and community environments that encourage physical activity among adolescents and reduce sedentary behavior are important for preventing obesity, developing physical health, and improving quality of life. Although the current study has provided valuable information about the level of physical activity and sedentary behavior in LMICs, the current analysis has several limitations due to its reliance on the conclusions of existing studies. The different methods and measurement indicators adopted in these studies may lead to certain biases in the comparison and analysis of results (Aubert et al., 2021). In addition, the analysis may not fully consider the specific circumstances of different countries and regions, such as policy, culture, and socioeconomic conditions. To gain a deeper understanding of the current state of physical activity and sedentary behavior, Darfour-Oduro et al. (2018) suggest that future studies could use a more consistent and standardized methodology and could survey a wider range of countries and regions. In addition, the factors that potentially affect physical activity and sedentary behavior can be studied to develop targeted interventions. This could include improving urban planning and public transport facilities, promoting physical education, and raising awareness about healthy lifestyles.

According to the adolescent PA and SB data provided by the GSHS, the incidence of insufficient PA and elevated SB in adolescents is very high (Durkee et al., 2016), and the characteristics differ according to sex and age. Interventions should be targeted to reduce SB based on gender and age because sedentary behavior and negative health outcomes have often been shown to be independently associated (Guthold et al., 2020). Due to the different physiological functions of men and women, girls are usually good at participating in low-intensity but durable sports, while boys are more interested in high-intensity sports. There are also differences in the types of exercise performed at different ages, and as the proportion of screen-related sedentary behavior increases with age, sedentary behavior in reading and learning fluctuates (Guthold et al., 2020). It may be necessary to promote other leisure activities, limit screen time, and incorporate sports breaks into students’ daily routines. It is concerning that these studies highlight significant gender and age differences, with girls being less active than boys and PA levels declining with age. These findings underscore the importance of developing targeted interventions and encouraging active lifestyles to improve overall health. However, a limitation may be that while the negative health effects of low PA and high SB have been highlighted, the interventions available to schools and physical education teachers have not been widely discussed (Hallal et al., 2012). Based on these findings, future research and action should focus on the details of interventions, such as how to create supportive environments for PA starting from the standard of safe sports space or how to maintain good sports facilities. Long-term observation and comparison experiments can be conducted to determine how physical education teachers can stimulate students’ motivation to live a healthy life, which can compensate for the shortcomings of previous studies that have tended to be theoretical and lacked implementation (Calestine et al., 2017). Schools should also play a central role in promoting PA by implementing a comprehensive physical education curriculum and providing extracurricular activities that cater to students’ diverse interests and abilities. More importantly, discussions on interventions need to focus on addressing gender and age differences in PA levels. Future research should to customize goals and strategies according to the unique needs of different population groups, especially to collect data on girls and older adolescents’ participation in PA because these two groups have usually been ignored in previous comparative studies (Hallal et al., 2012).

Gender differences in physical activity and sedentary behavior have been studied extensively. Peltzer & Pengpid (2016) suggested that girls from families with higher economic backgrounds are more likely to exhibit sedentary behavior, while boys, especially those from families with lower economic backgrounds, are more likely to be insufficiently physically active. This study provides comprehensive evidence for the link between gender differences, economic level and physical activity. Moreover, Ashdown-Franks et al. (2019b) confirmed the positive relationship between the frequency of drug use and sedentary behavior. However, the link between diet and physical activity has not been widely discussed, possibly because these two factors have a more direct impact on an individual’s health status, while the impact of physical activity is less significant (Vancampfort et al., 2019a). On the other hand, income level has proven to be a key factor that influences physical activity, although the effect of occupation type on sedentary behavior has not been discussed. Although some studies have begun to explore how culture influences physical activity and sedentary behavior, relatively little research has been conducted in this area. Issues such as whether consumer culture and celebrity influence may play a guiding role in physical activity and whether community culture can motivate regional residents warrant further exploration. Psychological factors, such as motivation and self-efficacy, may also affect individuals’ willingness to participate in sports activities (Zheng et al., 2023), and community environmental factors such as the availability of sports facilities may alter the effectiveness of sports (Liu et al., 2023). Therefore, based on the results from LMICs, it is necessary to comprehensively consider individual income, gender, and diet structure to optimize the selection of sports activities. Moreover, the promotion and support of physical activity by society and the community are important interventions.

### Study strengths and limitations

The current review has several notable strengths. First, this study represents an inaugural attempt to consolidate information on PA and sedentary behavior among school-going adolescents from LMICs. Second, an exhaustive database search was performed without imposing constraints on publication dates. However, certain limitations must be acknowledged. These limitations primarily include the requirement for studies to be in English and the inability to entirely eliminate the possibility of publication bias. Moreover, due to the considerable diversity in outcome assessment and analytical methods among the included studies, conducting a meta-analysis was not feasible.

### Practical implications

The above results suggest that the GSHS provides comprehensive and authentic official data on students’ health behaviors and protective factors. Improving the psychological quality of primary and middle school students and promoting the harmonious development of physical and mental health are the keys to further strengthening education and comprehensively improving its quality (Yan et al., 2023). With the development of physiology and psychology, the expansion of social experience, and changes in thinking, especially given the pressure of social competition, people encounter problems in adjusting interpersonal communication and continuing to learn. Analysis of the GSHS can help students develop positive psychological qualities through professional psychological education. Positive psychology is related to human growth and is the key to perseverance in the face of difficulties. The introduction of the GSHS in LMICs can help in exploring the protective factors for local adolescents’ mental health and can provide sufficient and effective resources for the establishment of adolescent health programs and related policies in LMICs. Moreover, the GSHS allows international agencies, countries, and other agencies to compare the health behaviors and protective factors among countries, which can help LMICs learn from the solutions of other countries when similar problems are encountered among children and adolescents to improve relevant policies and legal provisions. Finally, the GSHS predicts the development trend of adolescent health behaviors and protective factors in various countries according to the collected data, helps LMICs evaluate their adolescent health promotion programs, analyzes the reasons for these trends according to the physical condition of students in these areas, and proposes corresponding countermeasures. These countermeasures are highly practical for improving physical education and students’ physical quality in LMICs and are conducive to promoting the development of theoretical knowledge and practical activities related to teenagers’ mental health for parents, schools and society. In summary, the introduction of the GSHS in low- to middle-income countries can not only increase people’s attention to the mental health development of teenagers and reduce the occurrence of adolescent risk behaviors but also improve the use of relevant technologies, resources, and funds. Additionally, the survey methods and the professionalism of investigators can be improved, which would lay a good foundation for the reliability of the data and the development of the GSHS in the future.

## Conclusion

The amalgamated evidence from 29 studies in this review emphasizes that in comparison with high-income countries, adolescents in LMICs are still in the initial phase of understanding physical activity and are characterized by low levels of PA. Moreover, PA and sedentary behavior among school-going adolescents in LMICs are influenced by various factors, including policies, cultural norms, socioeconomic conditions, gender and age. Future research should prioritize aiding low- and middle-income countries in assessing their own adolescent health promotion programs. This analysis should be tailored to the specific physical conditions of students in each region, and corresponding measures should be proposed. This approach holds significant practical importance for enhancing physical education and improving the physical well-being of students in low- and middle-income countries. It also contributes to advances in theoretical knowledge in this field.

## Supplemental Information

10.7717/peerj.17097/supp-1 Supplemental Information 1 Search strategy.

10.7717/peerj.17097/supp-2 Supplemental Information 2 PRISMA checklist.

10.7717/peerj.17097/supp-3 Supplemental Information 3 other.

## Additional Information and Declarations

### Data Availability

The following information was supplied regarding data availability: The raw data is available in the tables.
