# Supplementary material for: Physical activity and sedentary behavior among school-going adolescents in low- and middle-income countries: insights from the global school-based health survey
Source: PeerJ. 2024 Apr 24;12:e17097. doi: 10.7717/peerj.17097 (PMC11055511; doi:10.7717/peerj.17097)
Supplement: Supplemental Information 1 [file peerj-12-17097-s001.docx]

Searching time：2023.03.18

database：WOS

Search strategy：AB=(global school-based student health survey) AND AB=(low and middle income)

Results：87 items

Searching time：2023.03.18

database：Ebsco host

Search strategy：AB global school-based student health survey AND AB ( low and middle income )

Results：85 items

Searching time：2023.03.18

database：ScienceDirect

Search strategy：Title, abstract, keywords: (global school-based student health survey) AND (low and middle income)

Results：43 items

Searching time：2023.03.18

database：Pubmed

Search strategy：(global school-based student health survey[Title/Abstract]) AND (low[Title/Abstract] AND middle income[Title/Abstract])

Results：74 items
