# Supplementary material for: Physical activity and sedentary behavior among school-going adolescents in low- and middle-income countries: insights from the global school-based health survey
Source: PeerJ. 2024 Apr 24;12:e17097. doi: 10.7717/peerj.17097 (PMC11055511; doi:10.7717/peerj.17097)
Supplement: Supplemental Information 3 [file peerj-12-17097-s003.docx]

Unfortunately, evidence linking physical activity and sedentary behavior in school-going adolescents from low- and middle-income countries is rare, but it is worth studying. To fill the research gap, this study, therefore, primarily aims to synthesize the evidence about physical activity and sedentary behavior in school-going adolescents from low- and middle-income countries based on the Global Student-based Health Survey.
